# FtlA and FtlB Are Candidates for Inclusion in a Next-Generation Multiantigen Subunit Vaccine for Lyme Disease

**DOI:** 10.1128/iai.00364-22

**Published:** 2022-09-14

**Authors:** Andrew C. Camire, Nathaniel S. O’Bier, Dhara T. Patel, Nicholas A. Cramer, Reinhard K. Straubinger, Edward B. Breitschwerdt, Rebecca A. Funk, Richard T. Marconi

**Affiliations:** a Department of Microbiology and Immunology, Virginia Commonwealth University Medical Center, Richmond, Virginia, USA; b Institute of Infectious Diseases and Zoonoses, Department of Veterinary Sciences, Faculty of Veterinary Medicine, Ludwig-Maximilians-University, Munich, Germany; c Department of Clinical Sciences, The Comparative Medicine Institute, College of Veterinary Medicine, North Carolina State University, Raleigh, North Carolina, USA; d Department of Large Animal Clinical Sciences, VA-MD College of Veterinary Medicine, Blacksburg, Virginia, USA; e Department of Oral and Craniofacial Molecular Biology, Philips Institute for Oral Health Research, School of Dentistry, Virginia Commonwealth University, Richmond, Virginia, USA; University of California, Davis

**Keywords:** *Borreliella*, *Borrelia*, BBK01, BBG01, Ftl, Lyme disease vaccine, protein family 12, FtlA, *Ixodes*, Lyme vaccine, PF12, canine Lyme disease, chimeritope

## Abstract

Lyme disease (LD) is a tick-transmitted bacterial infection caused by Borreliella burgdorferi and other closely related species collectively referred to as the LD spirochetes. The LD spirochetes encode an uncharacterized family of proteins originally designated protein family twelve (PF12). In B. burgdorferi strain B31, PF12 consists of four plasmid-carried genes, encoding BBK01, BBG01, BBH37, and BBJ08. Henceforth, we designate the PF12 proteins family twelve lipoprotein (Ftl) A (FtlA) (BBK01), FtlB (BBG01), FtlC (BBH37), and FtlD (BBJ08). The goal of this study was to assess the potential utility of the Ftl proteins in subunit vaccine development. Immunoblot analyses of LD spirochete cell lysates demonstrated that one or more of the Ftl proteins are produced by most LD isolates during cultivation. The Ftl proteins were verified to be membrane associated, and nondenaturing PAGE revealed that FtlA, FtlB, and FtlD formed dimers, while FtlC formed hexamers. Analysis of serum samples from B. burgdorferi antibody (Ab)-positive client-owned dogs (*n* = 50) and horses (*n* = 90) revealed that a majority were anti-Ftl Ab positive. Abs to the Ftl proteins were detected in serum samples from laboratory-infected dogs out to 497 days postinfection. Anti-FtlA and FtlB antisera displayed potent complement-dependent Ab-mediated killing activity, and epitope localization revealed that the bactericidal epitopes reside within the N-terminal domain of the Ftl proteins. This study suggests that FtlA and FtlB are potential candidates for inclusion in a multivalent vaccine for LD.

## INTRODUCTION

Lyme disease (LD) is a tick-transmitted infection (1, 2) caused by species of the genus Borreliella (formerly classified as Borrelia) (3). The primary pathogenic species in North America is Borreliella burgdorferi. In Europe, B. burgdorferi, Borreliella garinii, Borreliella afzelii, and Borreliella bavariensis are associated with disease (reviewed in reference 4). We refer to pathogenic Borreliella species collectively as the LD spirochetes. LD spirochetes are maintained in nature in an enzootic cycle involving Ixodes species ticks and diverse vertebrate reservoirs (5, 6). The CDC reported in 2016 that Ixodes scapularis and Ixodes pacificus ticks, the primary vectors for LD in North America, are present in 49.2% of United States counties (7). Since that report, the endemic regions for I. scapularis ticks have expanded, with overwintering populations recently confirmed in Douglas, Sarpy, and Saunders counties in Nebraska (8). It is estimated that there are at least 470,000 clinician-diagnosed cases of LD in humans each year in the United States alone (9), with similar numbers in Western Europe (10). The Companion Animal Parasite Council (https://capcvet.org/articles/parasite-prevalence-maps/), which tracks serological test results for tick-borne pathogens in client-owned dogs, reported 434,000 positive B. burgdorferi Ab-based tests in 2021 in the United States. It is important to note that since CAPC collects data from approximately 30% of the Ab tests that are run, the actual number of positive B. burgdorferi Ab tests is likely to be much greater.

The organization of the LD spirochete genome and that of the closely related relapsing fever spirochetes is unique among bacteria (11–15). The genome is segmented and comprised of an ~900-kb linear chromosome and a variable group of linear and circular DNA plasmids that constitute approximately 40% of the total DNA (11, 16, 17). The majority of B. burgdorferi surface proteins are encoded by gene families that are distributed among the plasmids (18). In this study, we investigate the expression, antigenicity, and immunogenicity of B. burgdorferi strain B31 protein family twelve (PF12) members BBK01, BBG01, BBH37, and BBJ08 (18), henceforth referred to as family twelve lipoprotein (Ftl) A (FtlA), FtlB, FtlC, and FtlD, respectively. The *ftlA, ftlB, ftlC* and *ftlD* genes of B. burgdorferi B31 are carried by the linear plasmids (lp) lp36 (plasmid K), lp28-2 (plasmid G), lp28-3 (plasmid H), and lp38 (plasmid J), respectively (19). The original annotation of PF12 included the chromosomally encoded open reading frame (ORF) BB0844 (18). B0844 shares only ~28% amino acid (aa) identity with other Ftl proteins. Hence, given its divergence and reports that it is dispensable for infection of ticks and mammals (20), BB0844 was not analyzed as part of this study. Beyond the information obtained from genome-wide studies, the Ftl proteins are largely uncharacterized (21–26). Here, we demonstrate that the Ftl proteins are expressed *in vitro*, antigenic during infection, membrane localized, surface exposed, and oligomeric. Abs generated in rats against FtlA and FtlB displayed potent complement-dependent bactericidal activity, whereas Abs against FtlC and FtlD did not. The bactericidal epitopes of FtlA were localized within its N-terminal region. The results suggest that FtlA and FtlB, or fragments thereof, may be of utility in the development of a next-generation multiantigen subunit vaccine for human and veterinary LD.

## RESULTS

### Ftl phylogeny.

**FIG 1:**
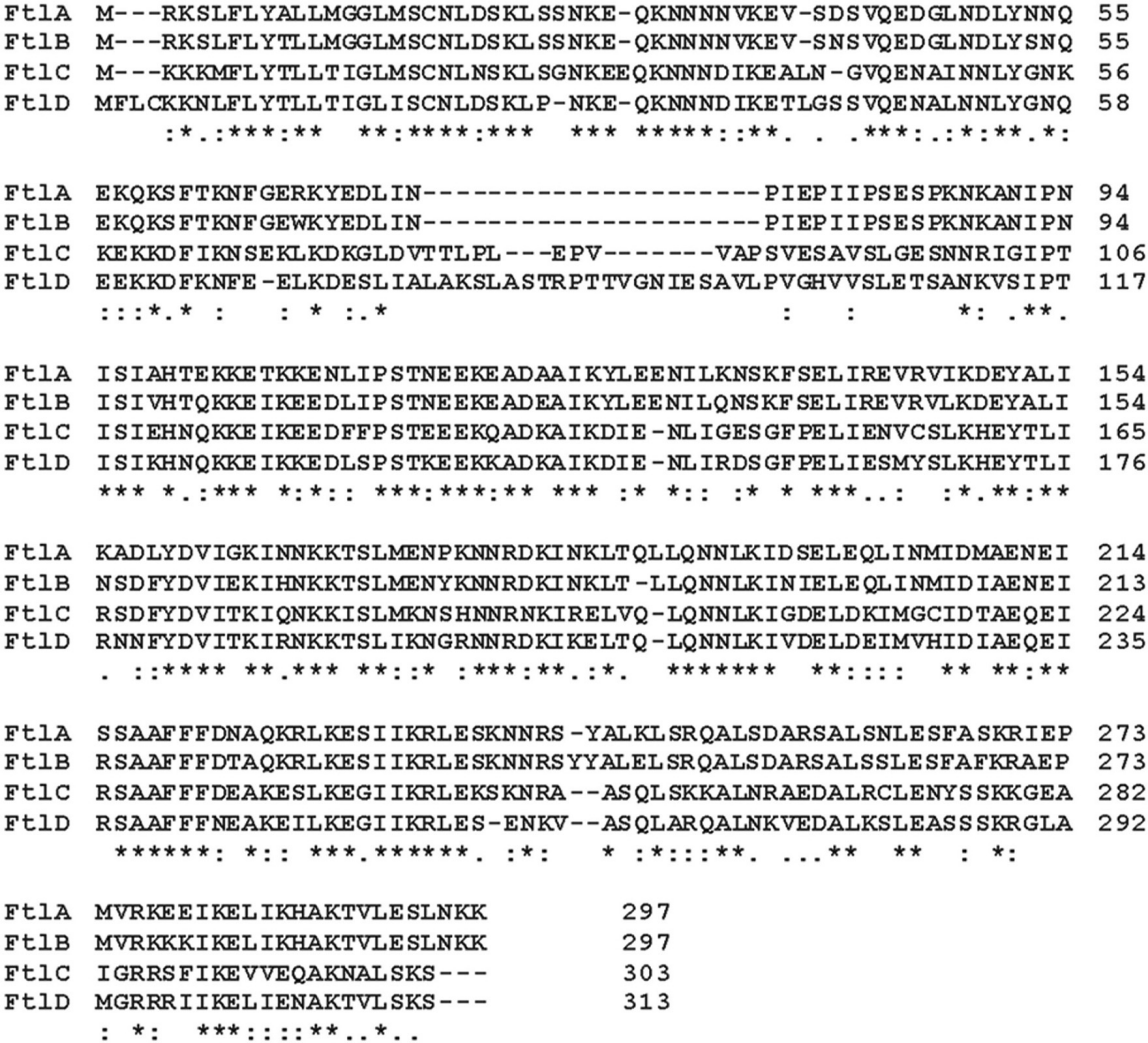
Amino acid sequence alignment of family twelve lipoproteins (Ftl). B. burgdorferi strain B31 FtlA, -B, -C, and -D sequences were aligned using Clustal Omega.

Using the FtlA sequence as the query in a BLASTP search, proteins with amino acid identity values of 89% or greater (100% query coverage) were detected in most B. burgdorferi genome sequences. Ftl orthologs were also detected in B. afzelii, B. garinii, B. bavariensis, other LD spirochete species (≤75% amino acid identity; ≥90% query coverage), and in the tick-borne relapsing fever Borrelia (≤40% amino acid identity; ≥98% query coverage). Alignment of the B. burgdorferi B31 Ftl amino acid sequences identified insertions/deletions and polymorphisms in the N-terminal domain of the proteins (Fig. 1). Percent amino acid similarity and identity values are presented in Table 1. FtlC and FtlD harbor insertions that are not present in FtlA or FtlB. BLASTP searches using the FtlC and FtlD insertions revealed that these sequences are present in only a limited number of LD isolates.

**TABLE 1:** B. burgdorferi Ftl amino acid identity and similarity values

Protein	% amino acid identity (lower left quadrant) or similarity (upper right quadrant) of:			
	FtlA (34.3 kDa)	FtlB (34.6 kDa)	FtlC (34.3 kDa)	FtlD (34.6 kDa)
FtlA		89.6	52.5	53.8
FtlB	96.3		55.1	55.8
FtlC	81.5	81.8		68.4
FtlC	77.6	77.9	87.5	

### Immunological cross-reactivity of Abs to the Ftl proteins.

**FIG 2:**
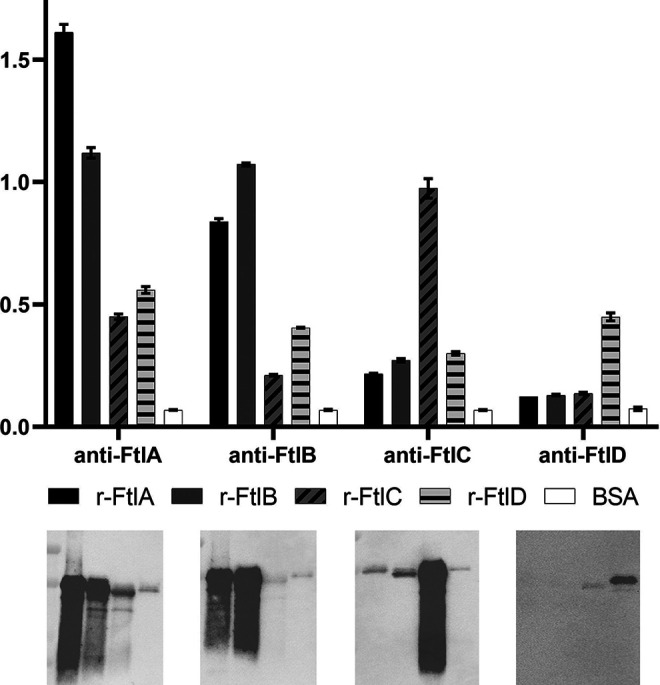
Analysis of the specificity of anti-Ftl antisera. The immunoreactivity of recombinant FtlA (r-FtlA), r-FtlB, r-FtlC, and r-FtlD with antiserum raised against each protein was assessed by ELISA and immunoblotting. BSA served as the negative-control immobilized protein in the ELISA analyses. The antisera used in the assays are indicated along the *x* axis, and the *y* axis indicates the absorbance measured at 405 nm. ELISAs were run in triplicate. The recombinant Ftl proteins were also screened using an immunoblot format with each antiserum (from left to right: anti-FtlA, anti-FtlB, anti-FtlC and anti-FtlD). As a negative control, an immunoblot was screened with preimmune serum (not shown).

To determine if the Ftl proteins shared conserved epitopes, the recombinant proteins were screened with serum generated against each Ftl paralog using both enzyme-linked immunosorbent assay (ELISA) and immunoblot formats (Fig. 2). Anti-FtlA and anti-FtlB antisera reacted strongly with both FtlA and FtlB, indicating shared epitopes. Anti-FtlC and anti-FtlD antisera reacted with the homologous protein but weakly with the other Ftl proteins.

**FIG 3:**
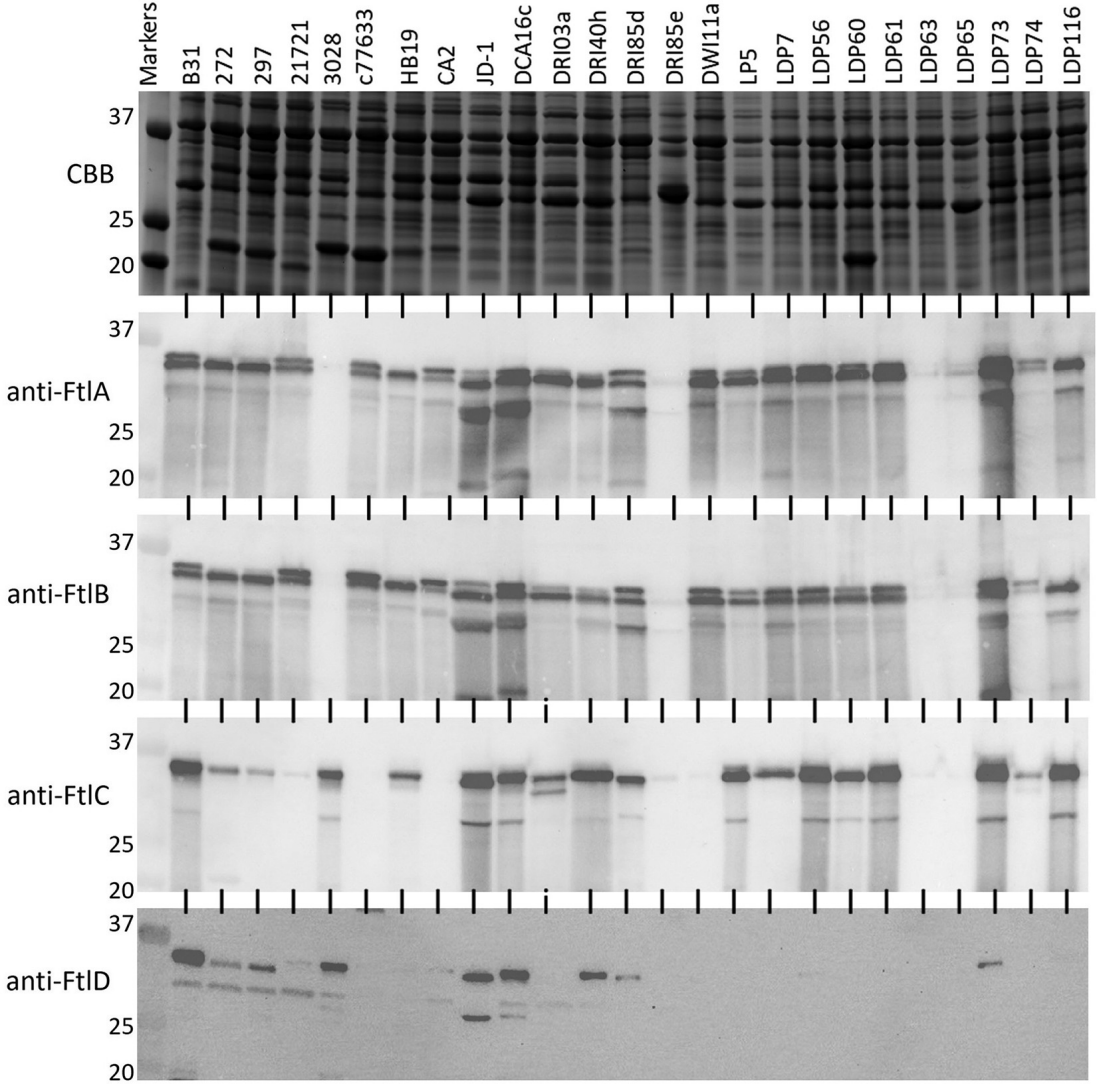
Ftl expression by diverse B. burgdorferi isolates during cultivation. Cell lysates of 25 B. burgdorferi isolates from North America (indicated along the top) were fractionated by SDS-PAGE, and the proteins stained with Coomassie brilliant blue (CBB) (top) or transferred to PVDF membranes. Identical immunoblots were screened with antiserum (1:1,000 dilution) as indicated to the left. MW standards are indicated on the left (in kDa). Cell lysates of European LD isolates were screened, and the results are presented in Fig. S1.

To assess Ftl production during cultivation, cell lysates of LD isolates from the United States and Europe were immunoblotted and screened with anti-FtlA, anti-FtlB, anti-FtlC, and anti-FtlD antisera (Fig. 3; Fig. S1 in the supplemental material). One or more immunoreactive proteins were detected with the antisera in 83% of the isolates tested. The immunoreactive profiles of B. burgdorferi cell lysates screened with anti-FtlA and anti-FtlB antisera were nearly identical, indicating that they harbored conserved epitopes. While the molecular weights (MWs) of FtlA (34.3 kDa) and FtlB (34.6) were similar, two bands were distinguishable in most isolates. Among European LD isolates, the immunoreactive patterns with anti-FtlA and anti-FtlB antisera were identical, but they differed from those of North American B. burgdorferi isolates. Only a single dominant immunoreactive band was observed with the FtlA and FtlB antisera, and the MWs of the immunoreactive proteins varied significantly among isolates. The immunoreactive patterns observed with anti-FtlC and anti-FtlD antisera differed from those detected with anti-FtlA and anti-FtlB antisera. Some isolates that were positive for FtlA and/or FtlB were negative for FtlC and vice versa. Proteins that were immunoreactive with the anti-FtlD antiserum were detected in only a few of the isolates tested. In some cell lysates, proteins smaller in size than known Ftl proteins were detected. Examples include B. burgdorferi JD1, DCA16c (Fig. 3), and VS134 and B. garinii Jem5 (Fig. S1). The potential basis for these polymorphisms is discussed below. It is important to note that due to the similar molecular weights of the Ftl proteins and their immunological cross-reactivity, the immunoblot analyses did not allow definitive identification of the proteins as FtlA or FtlB.

### Ftl proteins form oligomers and localize to the outer membrane.

**FIG 4:**
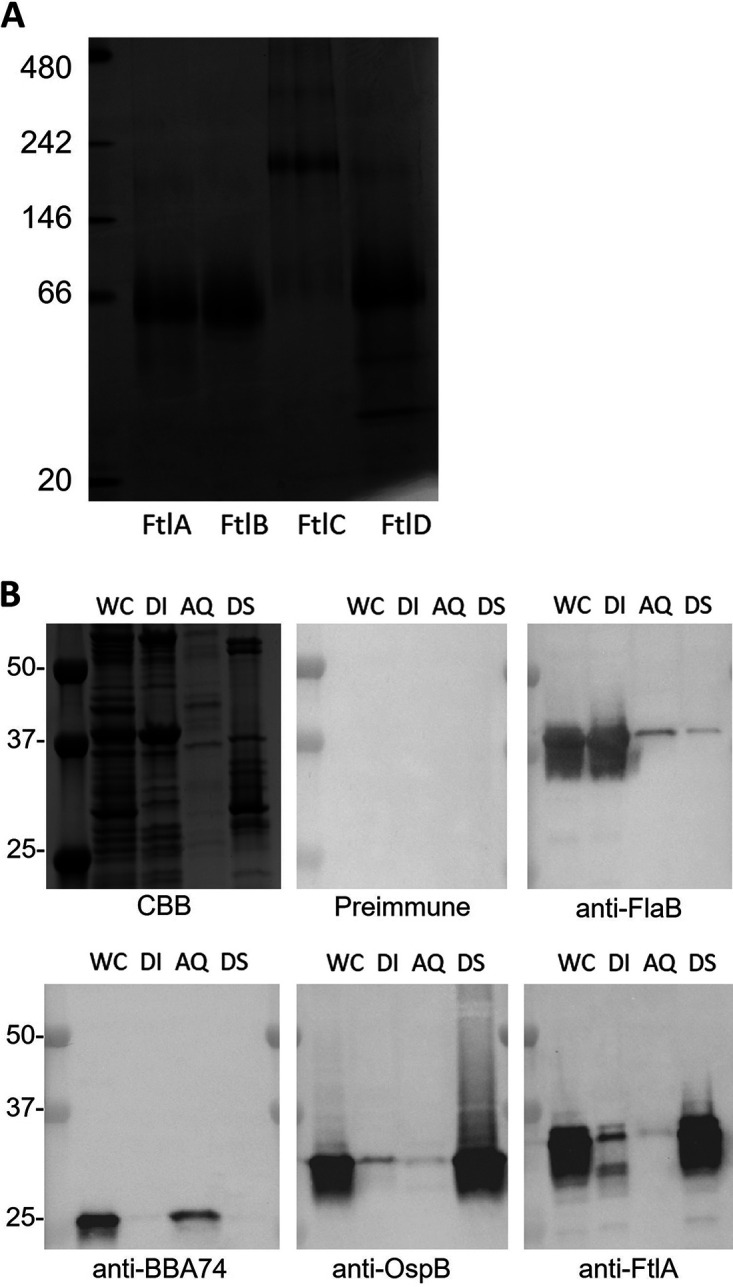
The Ftl proteins are oligomeric and localize in the outer membrane. (A) Recombinant Ftl proteins were fractionated by BN-PAGE and visualized by staining with CBB. MW markers are shown to the left. (B) To assess subcellular localization, B. burgdorferi B31 cells were subjected to Triton X-114 extraction and phase partitioning, and the fractions were analyzed by SDS-PAGE, stained with CBB or transferred to PVDF membranes, and screened as indicated below each panel. WC, whole-cell lysate; DI, detergent-insoluble phase; AQ, aqueous phase; DS detergent-soluble phase.

The oligomeric state of recombinant Ftl proteins was assessed using blue native (BN)-PAGE. FtlA, FtlB, and FtlD existed in solution predominantly as dimers (apparent MW of 66 kDa), whereas FtlC was primarily hexameric (Fig. 4A). To assess cellular localization, Triton X-114 extraction and phase partitioning were performed with B. burgdorferi B31 cells, and the resulting fractions were immunoblotted and screened with anti-FtlA antiserum (Fig. 4B). Immunoreactive proteins were detected exclusively in the detergent-soluble (DS) fraction, indicative of membrane localization. Controls for cell localization included immunoblots screened for FlaB (inner membrane-anchored periplasmic protein), OspB (outer surface membrane lipoprotein), and BBA74 (periplasmic protein) (27). FlaB was detected specifically in the detergent-insoluble (DI) (protoplasmic cylinder) fraction, OspB in the detergent-soluble phase (DS), and BBA74 in the aqueous (AQ) phase. It can be concluded that the Ftl proteins are membrane associated.

### Ftl antigenicity during infection in dogs and horses.

**FIG 5:**
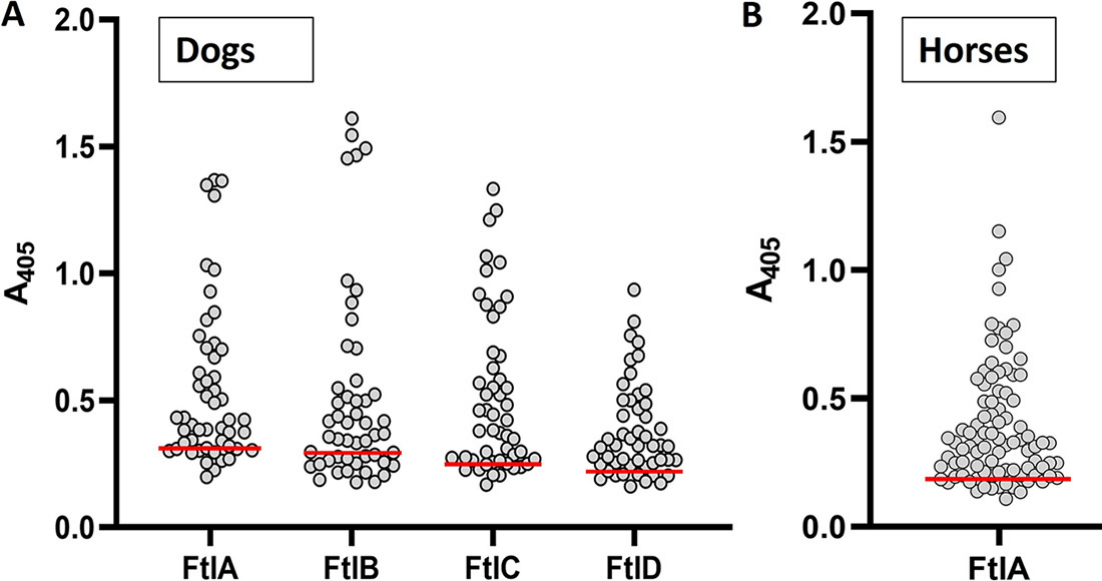
Ftl proteins are antigenic during infection. (A) Recombinant Ftl proteins were immobilized in ELISA plate wells and screened with sera (1:1,000) from B. burgdorferi Ab-positive dogs (*n* = 50). (B) Sera from B. burgdorferi Ab-positive client-owned horses (*n* = 90) were screened for Abs to the Ftl proteins using FtlA as the immobilized antigen. Circles indicate the mean absorbance for each serum sample (triplicate analysis). Samples that yielded a mean *A*_405_ value 2-fold over the mean background absorbance upon screening with sera from healthy dogs or horses (red lines) were scored as Ab positive.

**FIG 6:**
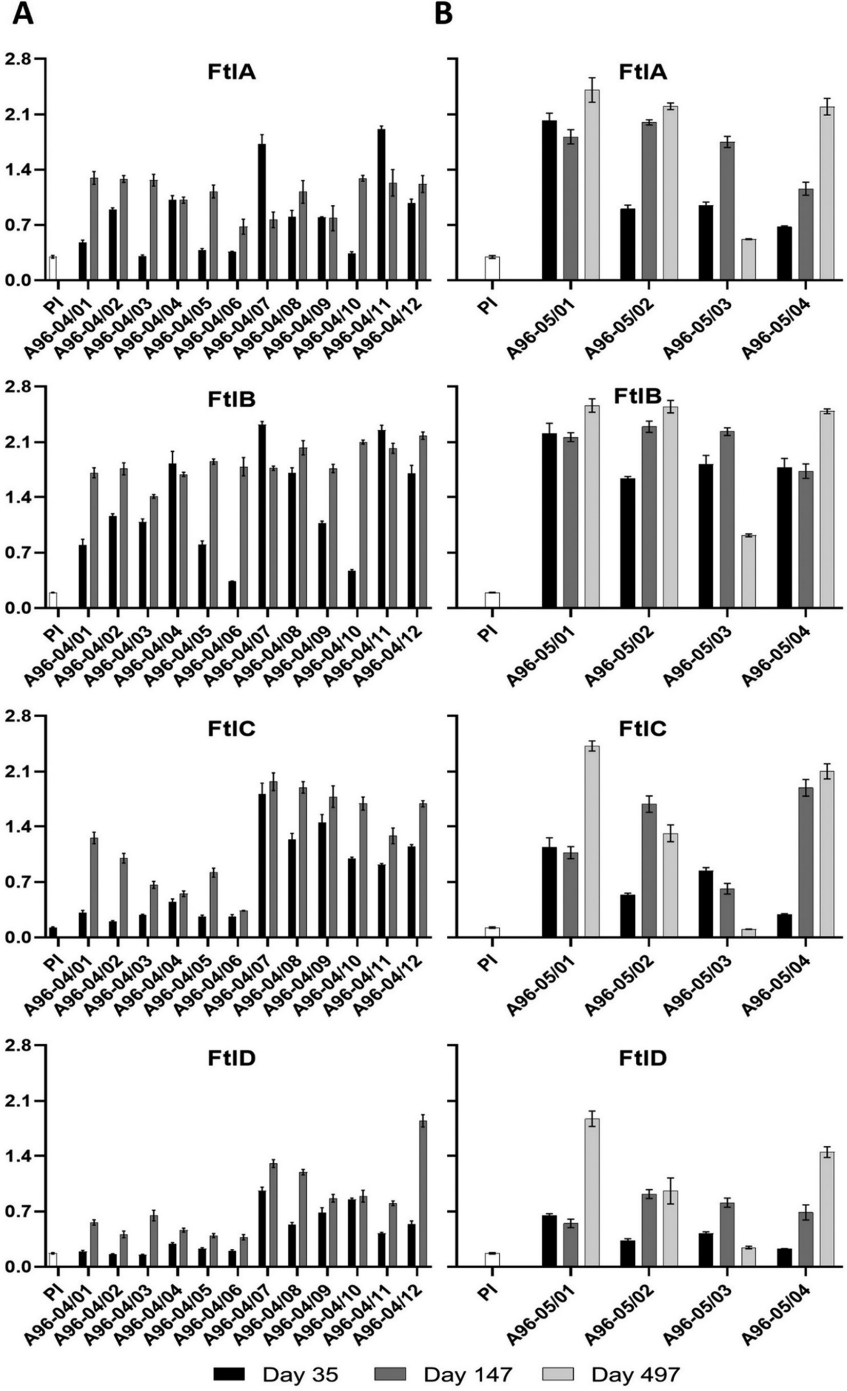
Ab screening of experimentally infected dogs. Sera collected from experimentally infected dogs were screened for Abs to the Ftl proteins using an ELISA format. (A) Serum samples collected on days 35 and 147 postinfection were screened. (B) A subset of the dogs that remained in the study for 497 days were screened for Abs to the Ftl proteins using serum samples collected on days 35, 147, and 497 post-infection feeding. Absorbance values at 405 nm are indicated on the *y* axis, and animal identification numbers are indicated on the *x* axis. Preimmune canine sera (PI) served as the negative control. Error bars represent the standard deviations from triplicate assays.

In an earlier study, Dowdell et al. speculated that FtlA and FtlB were required for B. burgdorferi persistence in vertebrates (28). To indirectly assess expression in mammals, serum samples from 50 B. burgdorferi C6 peptide Ab-positive client-owned dogs were screened for Abs to the Ftl proteins by ELISA (Fig. 5A). The percentages of serum samples that harbored Ftl-directed Abs ranged from 66% to 84% when FtlB and FtlC, respectively, were used as the detection antigens. Screening of horse serum samples using FtlA as the immobilized antigen revealed that 61% harbored Abs that bound to FtlA (Fig. 5B). To determine if Ftl proteins were produced throughout infection or if their expression was stage specific, dogs were infected via two successive rounds of tick feeding. Serum samples were collected 35 and 147 days postinfection and screened for anti-Ftl Abs (Fig. 6A). A general trend of elevated Ab levels over time was observed for most samples. A limited number of serum samples were available from dogs that were infected for 497 days. Consistent with the data described above, anti-Ftl Ab levels as determined by single-dilution ELISA analyses increased with time (Fig. 6B). The persistence of anti-FtlA Ab suggests that one or more Ftl proteins are expressed throughout infection.

### Comparative analysis of the bactericidal activities of Abs elicited by the Ftl proteins.

**FIG 7:**
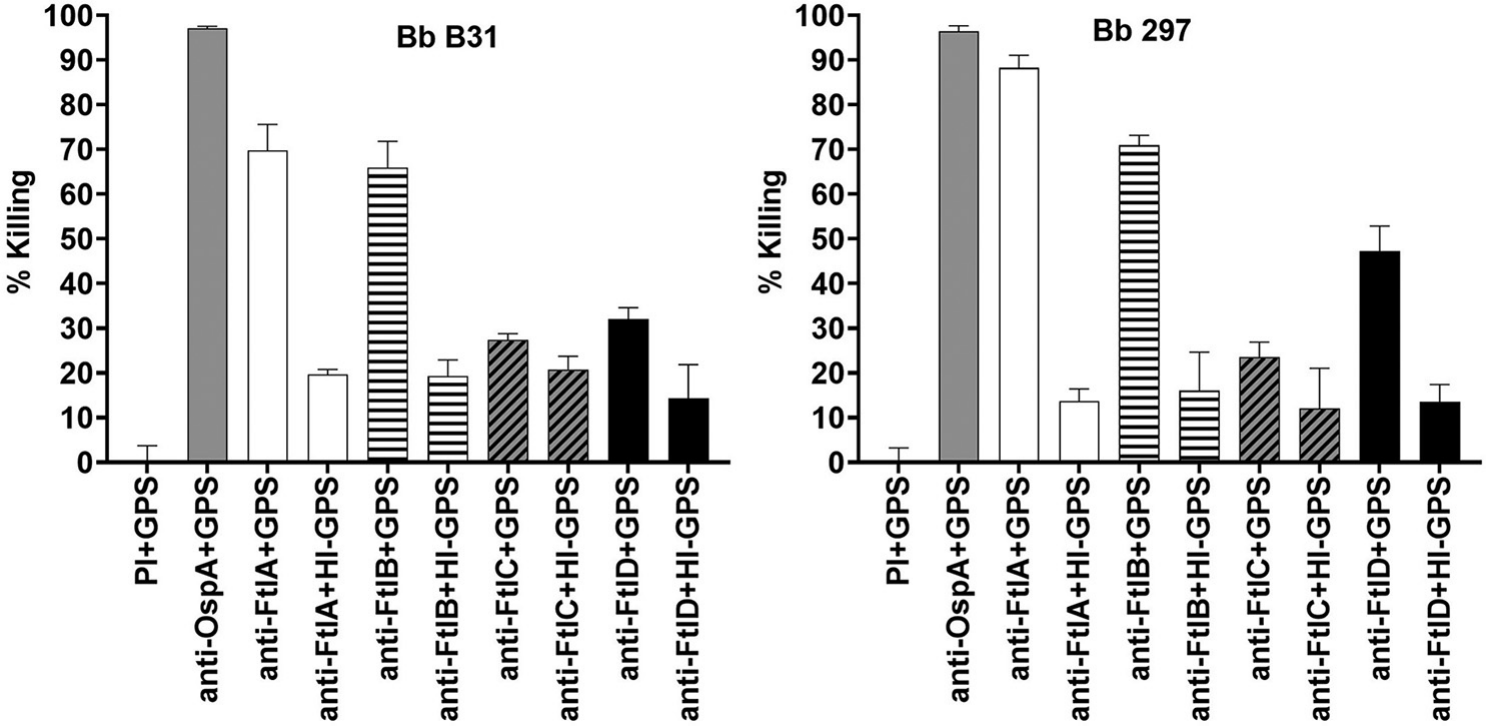
Comparative analysis of the bactericidal activities of anti-Ftl antisera against B. burgdorferi strains B31 and 297. Hyperimmune sera raised against FtlA, -B -C, and -D were tested for bactericidal activity against B. burgdorferi B31 and 297 in the presence of complement activity-certified guinea pig serum (GPS) or heat-inactivated (HI) GPS. Preimmune rat serum (PI) with GPS and anti-OspA antiserum with GPS served as the negative and positive controls, respectively, for bactericidal activity. Error bars show standard deviations.

To determine if anti-Ftl Abs had potential bactericidal activity, sera from rats immunized with FtlA, FtlB, FtlC, or FtlD were incubated with B. burgdorferi isolates B31 and 297 in the presence of complement activity-certified guinea pig serum (GPS) or heat-inactivated GPS (HI-GPS) (Fig. 7). Anti-OspA antiserum served as a positive control for bactericidal activity, and as expected due to the high level of OspA expression during cultivation, complete killing was observed. Anti-FtlA and anti-FtlB antisera displayed potent Ab-mediated, complement-dependent bactericidal activity against both test strains (>65%). In contrast, anti-FtlC and anti-FtlD antisera had low bactericidal activity. The lower levels of killing with these anti-Ftl antisera are consistent with the low levels of expression of FtlC and FtlD during cultivation.

### Localization of the immunodominant domain and bactericidal epitopes of FtlA.

**FIG 8:**
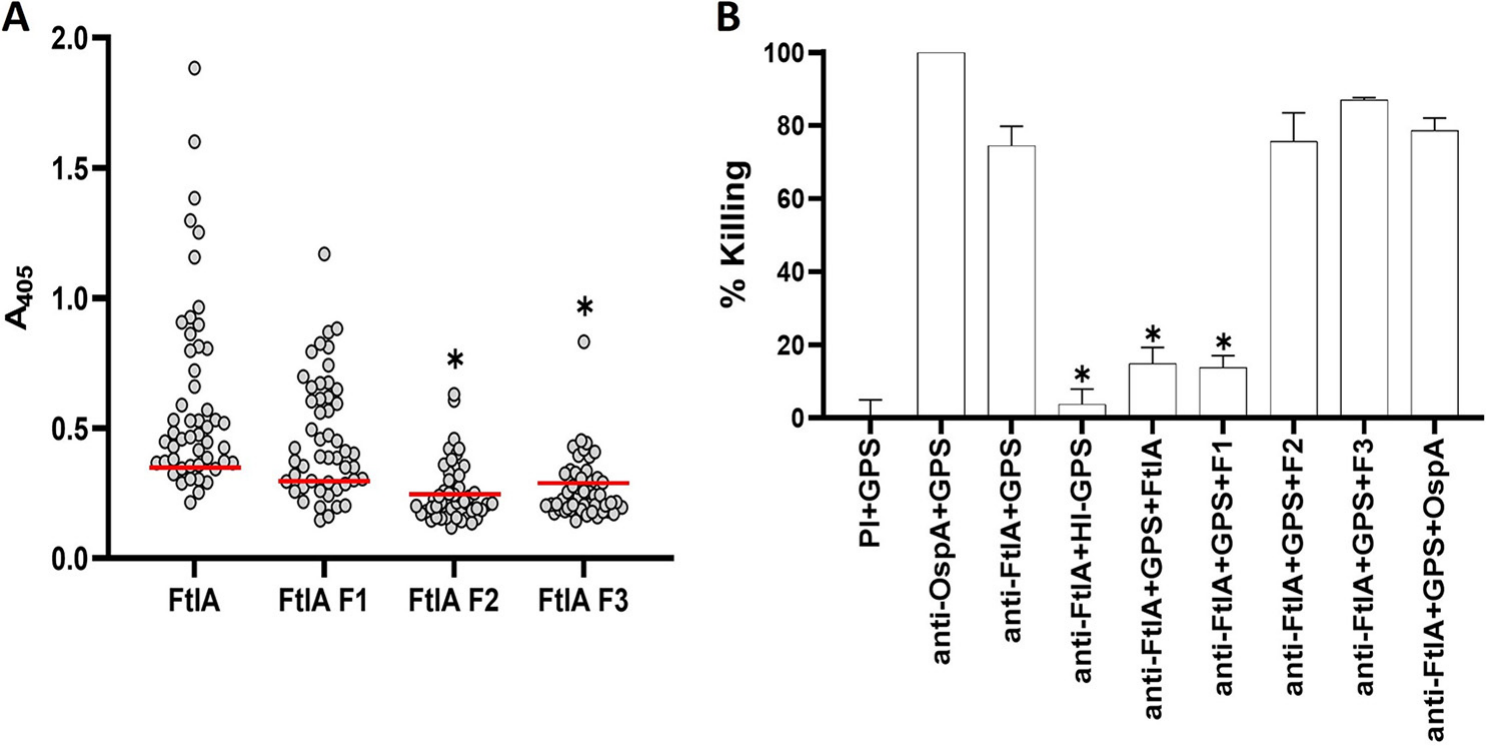
The immunodominant epitope that elicits Abs with bactericidal activity is located within the N-terminal segment of FtlA. (A) Recombinant full-length FtlA and three overlapping FtlA fragments (F1, F2, and F3) were screened with sera from B. burgdorferi Ab-positive client-owned dogs by ELISA (*n* = 50). Preimmune canine sera (PI) served as the negative control (data not shown). The horizontal red lines indicate the positive-threshold cutoff. Significance was determined by one-way ANOVA with 95% CIs, comparing mean absorbance readings of FtlA F2 and F3 to FtlA F1 (*P* < 0.0001). (B) To determine if FtlA and the F1, F2, and F3 fragments inhibit Ab-mediated killing of B. burgdorferi B31, anti-FtlA antiserum was incubated with each protein prior to its addition to live cells. Percent killing was calculated as detailed in Materials and Methods. Significance was determined by one-way ANOVA with 95% CIs, comparing each test condition to the results for anti-FtlA antiserum with GPS (*P* < 0.0001). Error bars show standard deviations. *, *P* < 0.05.

To identify the immunodominant region of FtlA, full-length FtlA and overlapping fragments spanning the length of the protein (F1, F2, and F3) were screened with B. burgdorferi peptide C6 Ab-positive serum samples from client-owned dogs (Fig. 8A). Of the 50 dogs screened, 74% (37/50) were Ab positive for fragment F1, whereas only 44% and 28% were Ab positive for the F2 and F3 fragments, respectively. The results indicate that the immunodominant epitopes of FtlA and FtlB are localized within the N-terminal domain of each protein.

To determine if the bactericidal epitope(s) of FtlA reside within its immunodominant N-terminal domain, bactericidal assays were performed in which anti-FtlA antiserum was incubated with full-length FtlA and fragments F1, F2, and F3 prior to mixing with cells and GPS (Fig. 8B). Full-length FtlA and F1 inhibited Ab-mediated killing, whereas the F2 and F3 fragments did not. As a negative control, the anti-FtlA antiserum was incubated with recombinant B. burgdorferi B31 OspA prior to mixing with cells, and as expected, OspA did not block anti-Ftl Ab-mediated complement-dependent killing. It can be concluded that the dominant epitopes that elicit bactericidal Abs are contained within the N-terminal domain of FtlA.

## DISCUSSION

In this report, we demonstrate that while the *ftl* gene family is widely distributed among Borreliella isolates, not all *ftl* genes are universal. While most isolates produce FtlA and/or FtlB during cultivation, only a subset express FtlC or FtlD. Consistent with the high percent amino acid sequence identity values of B. burgdorferi strain B31 FtlA and FtlB (89.6% identity and 96.3% similarity), the immunoblot profiles observed upon screening Borreliella cell lysates with anti-FtlA and anti-FtlB antisera were nearly identical. It is evident that FtlA and FtlB share conserved epitopes, and analysis of their sequences suggests that they map within N-terminal domain residues 54 to 94 (Fig. 1). This domain is conserved in FtlA and FtlB but divergent from the sequences of FtlC and FtlD and, thus, likely accounts for the specificity of the Ab response. FtlC and FtlD also harbor unique insertion sequences in their N-terminal domains of 11 (TTLPLEPVVAP) and 21 (PAKSLASTRPTTVGNIESAVL) amino acids, respectively. The FtlD insertion sequence is found in only a few of the annotated Ftl protein sequences, and it is unique to B. burgdorferi. The FtlC insertion sequence is more widely distributed among B. burgdorferi isolates and is present in some B. garinii Ftl proteins.

The immunoblot analyses revealed variation in the MWs of the Ftl proteins among isolates. It is notable that *ftlA* (BBK01), *ftlB* (BBG01), and *ftlC* (BBH37) are located near linear-plasmid telomeres (18), which are high-frequency sites for recombination (29–32). To determine if the absence of FtlA and FtlB in some isolates was the result of recombination or loss of plasmid K, the isolates were screened for BBK19 by immunoblot analysis (Fig. S2). BBK19 is encoded by the same plasmid as FtlA, but its gene maps to the center of plasmid K. The FtlA-negative isolates LDP63, LDP65, and DRI85e were positive for BBK19 by both immunoblotting and PCR (data not shown). However, we also observed the opposite. Isolates c77633 and LD7 were positive for FtlA but negative for BBK19, and isolate 3028 was negative for both FtlA and BBK19. Collectively, these data demonstrate that plasmid profile polymorphisms that may have resulted from recombination or plasmid loss influence the composition of the *ftl* gene family among isolates.

Triton X-114 extraction and phase partitioning demonstrated that the Ftl proteins are membrane associated, and BN-PAGE revealed that FtlA, FtlB, and FtlD exist in solution as dimers. Interestingly, FtlC is distinct from other Ftl proteins in that it predominantly forms hexamers. The biological significance of this higher-order FtlC oligomer remains to be determined. Brangulis et al. have recently determined the atomic structure of FtlA and demonstrated it to be a dimer (K. Brangulis, personal communication). While the sequences for FtlB and FtlD were structurally superimposable with the sequence of FtlA, the sequence of FtlC was not. This further indicates that FtlC is structurally distinct from other Ftl proteins and may carry out a distinctly different biological function.

It is noteworthy that while FtlC and FtlD are not produced by all isolates during cultivation, a majority of the dog serum samples were positive for Abs that bound to FtlC and/or FtlD (84%). This suggests that the expression of FtlC and FtlD may be upregulated during infection in mammals. The lack of detection of individual Ftl proteins in some cultivated isolates could be due to differential transcriptional expression. Genome-wide transcriptome and proteome analyses have yielded conflicting results regarding the expression patterns of the Ftl proteins (21–23, 33, 34). Tokarz et al. reported 2-fold downregulation of FtlB, FtlC, and FtlD in B. burgdorferi bacteria cultivated in the presence of blood (26), and based on this finding, they suggested that *ftl* expression is downregulated during infection. However, as demonstrated here, the detection of Abs to Ftl proteins in a majority of animals is indicative of expression during infection and the detection of Abs 497 days after initial infection suggests that Ftl production is ongoing.

Based in part on the *in vivo* expression and antigenicity of the Ftl proteins, we assessed properties that are relevant to potential use as a vaccine antigen. Epitope localization studies demonstrated that the immunodominant domain of FtlA (and presumably that of FtlB) is localized within residues 19 to 143 (F1 fragment). This finding is consistent with BepiPred 2.0 B cell linear epitope analyses (35, 36) that predict an extended antigenic region in FtlA and FtlB spanning residues 19 to 120. Similarly, BepiPred also predicts extended antigenic regions in the N-terminal domain of FtlC and FtlD. To determine if the Ftl proteins elicit Abs with bactericidal activity, serum from immunized rats was incubated with B. burgdorferi strains B31 and 297, with and without active complement. Abs to FtlA and FtlB displayed potent complement-dependent bactericidal activity, while Abs to FtlC and FtlD did not. Based on the analyses described above, we sought to determine if the epitopes that elicited bactericidal activity were also located within the N-terminal domain of FtlA and FtlB. Preincubation of anti-FtlA antiserum with FtlA or FtlA F1 attenuated the bactericidal activity of the hyperimmune serum.

The inherent genetic and antigenic diversity and stage-specific expression of some LD spirochete antigens during the enzootic cycle has complicated efforts to develop broadly protective subunit vaccine formulations. Chimeric vaccine antigens offer a potential solution, as they can be designed to elicit Abs against multiple protein variants/targets from one or more pathogens that can be synergistic (37). Vanguard crLyme (Zoetis) (38, 39), which is widely used in veterinary medicine, was the first chimeric-epitope-based protein (chimeritope) vaccine to be commercially developed (38, 39). In this report, we provide data that support the use of the immunodominant domains of FtlA and FtlB as potential components of a multiantigen vaccine formulation. Efforts are under way to identify additional antigens that can be incorporated into a next-generation chimeric antigen subunit vaccine for LD.

## MATERIALS AND METHODS

### Bacterial cultivation.

Borreliella isolates (Table S1) were cultivated in Barbour-Stoenner-Kelly II (BSK-II) medium supplemented with 6% rabbit serum at 34°C (without gelatin). Growth was monitored using wet mounts and dark-field microscopy. Cells were harvested from late-log-phase cultures by centrifugation.

### Generation of recombinant proteins.

The B. burgdorferi strain B31 *ftlA* (encoding the protein with accession number WP_106017559.1), *ftlB* (WP_010890300.1), *ftlC* (WP_012672184.1), and *ftlD* (WP_146124603.1) gene sequences were codon optimized for expression in Escherichia coli, synthesized (minus the leader sequence), and inserted into pET45b(+) at its BamHI and EagI restriction sites (GenScript). Overlapping *ftlA* gene fragments corresponding to amino acids 19 to 143 (F1), 109 to 233 (F2), and 171 to 297 (F3) were PCR amplified from B. burgdorferi B31 DNA using *Phusion* polymerase, standard amplification conditions, and primers that harbor BamHI (forward primer) and EagI (reverse primer) restriction sites (40). The amplicons were cut with BamHI and EagI, purified, and ligated into pET45b(+) (Novagen), and the plasmids propagated in E. coli NovaBlue cells. The plasmids were purified and transformed into E. coli BL21(DE3) cells, protein expression was induced with IPTG (isopropyl-β-d-thiogalactopyranoside; 1 mM), and the His-tagged proteins were purified using nickel affinity chromatography on an ÄKTA fast protein liquid chromatography (FPLC) platform (Cytiva) (40). Some proteins were subjected to a second round of FPLC purification using a cobalt column on the ÄKTA platform.

### Generation of antisera and description of serum samples.

Antisera to FtlA, FtlB, FtlC, FtlD, FlaB, BBA74, OspA, and OspB were generated in Sprague-Dawley rats using recombinant purified proteins (25 μg) and a three-dose immunization protocol (each dose 3 weeks apart) (41). The initial dose was delivered in Freund’s complete adjuvant (Sigma-Aldrich) and the boosters in Freund’s incomplete adjuvant. Rats were sacrificed 1 week after the last dose, and blood was collected by cardiac puncture. Serum was harvested using Z serum sep clot activator columns (Greiner). Serum samples from B. burgdorferi-infected client-owned dogs were provided by the Vector Borne Diseases Diagnostic Laboratory, Department of Clinical Sciences, College of Veterinary Medicine, North Carolina State University. The samples were tested for Abs to the B. burgdorferi VlsE-derived C6 peptide using the Snap4 Dx plus test (IDEXX). Sera from dogs that had been experimentally infected with B. burgdorferi by two successive rounds of infestation with field-collected Ixodes scapularis ticks (Westchester County, New York, USA) were also available for analysis (42). Sera from client-owned horses resident in Virginia were provided by the Department of Large Animal Clinical Sciences, VA-MD College of Veterinary Medicine, Virginia Tech. All animal experiments were conducted following the *Guide for the Care and Use of Laboratory Animals* (43) and in accordance with protocols peer reviewed and approved by Virginia Commonwealth University Institutional Animal Care and Use Committees.

### SDS-PAGE and immunoblot analyses.

Cells were recovered by centrifugation and washed with phosphate-buffered saline (PBS), and cell lysates generated by sonication. The cell lysates and recombinant proteins were fractionated by SDS-PAGE using precast Any kD Criterion gels (Bio-Rad). Equal loading of proteins and cell lysates was confirmed by staining representative gels with Coomassie brilliant blue (CBB). The proteins were transferred to polyvinylidene difluoride (PVDF) membranes as previously described (41) for immunoblot analyses. Anti-Ftl antisera were used at a 1:1,000 dilution except where specifically noted. Horseradish peroxidase (HRP)-conjugated anti-rat IgG (ThermoFisher) was used at a 1:40,000 dilution. Ab binding was detected using Clarity Western ECL substrate and chemiluminescence (Bio-Rad). Images were captured using a Bio-Rad ChemiDoc imaging system (Bio-Rad). Some images were cropped to remove blank space to facilitate the generation of figures.

### Triton X-114 extraction and phase partitioning.

Triton X-114 extraction and phase partitioning were performed as previously described (44). Briefly, B. burgdorferi B31 cells were harvested by centrifugation, washed, suspended in PBS with 1% Triton X-114, and incubated overnight (4°C with gentle agitation), and the detergent-insoluble (DI) fraction was collected by centrifugation (15,000 × *g* and 4°C). The supernatant was removed, incubated at 37°C (15 min), and centrifuged to separate the aqueous (AQ) and detergent-soluble (DS) phases, and the process was repeated. The samples were fractionated by SDS-PAGE, transferred to PVDF membranes, and screened with anti-FtlA, anti-FlaB, anti-BBA74, and anti-OspB antisera as described above.

### Blue native (BN)-PAGE analysis.

Recombinant proteins (5 μg) were diluted in 1× NativePAGE sample buffer (Thermo Fisher) with 0.25 μL of NativePAGE 5% G250 sample additive (Thermo Fisher) and fractionated in precast NativePAGE 4-to-16% Bis-Tris 1.0-mm mini-protein gels according to the manufacturer’s protocol (150 V for 110 min; Thermo Fisher). NativeMark unstained protein standards (ThermoFisher) were used to determine the molecular weights of the Ftl protein complexes. After electrophoresis, the gels were stained with CBB and then destained (40% methanol, vol/vol, and 10% glacial acetic acid, vol/vol) for 2 to 3 h with gentle rocking. The buffer was changed every 10 min, and the gels were imaged using a ChemiDoc imaging system (Bio-Rad).

### ELISA analyses.

ELISA plate wells were coated with 500 ng of recombinant protein in bicarbonate buffer overnight at 4°C (45). Rat anti-FtlA, -B, -C, and -D antisera were used at a 1:80,000 dilution, and horse and canine sera at a 1:100 dilution. Secondary Abs were used at a 1:15,000 dilution. ABTS [2,2′-azinobis(3-ethylbenzthiazolinesulfonic acid)] substrate was added, the plates were incubated for 20 min, and the absorbance was measured at 405 nm. Preimmune serum served as a negative-control serum sample, and bovine serum albumin (BSA) served as the immobilized negative control for nonspecific Ab binding. A serum sample was scored as Ftl Ab positive if the mean absorbance value in the single-dilution ELISA analyses was 2-fold greater than the mean absorbance value of preimmune serum with each antigen.

### Bactericidal assays.

Bactericidal assays were conducted as previously described (41). In brief, mid-log-phase B. burgdorferi B31 cultures were incubated overnight with heat-inactivated (HI) (56°C for 30 min) anti-Ftl antisera. Cultures were combined with HI hyperimmune serum (final concentration, 20%), complement activity-certified guinea pig serum (GPS) (20%; Complement Tech), or HI-GPS (20%) and incubated. As controls, cells were incubated with preimmune (PI) serum (20%) with complement activity-certified GPS (20%) or in medium alone. The number of live cells in five fields of view was determined by visual counting of motile spirochetes using wet-mount dark-field microscopy. The values were averaged, and the percentage of killing was calculated by comparing the number of live cells after incubation with HI hyperimmune serum and GPS to the number after incubation with preimmune sera with GPS.

To localize the bactericidal epitopes of FtlA, Ab-blocking experiments were performed. HI anti-FtlA antiserum was incubated with or without 1 μg of FtlA, fragment F1, F2, or F3, or B. burgdorferi OspA (negative control) (20%) (2 h at 34°C) before combining with cells and complement-active GPS. Bactericidal activity was measured as detailed above. All assays were performed in triplicate with at least two biological replicates.

### Statistical analyses.

For ELISAs, significance was assessed using a one-way analysis of variance (ANOVA) with Tukey’s multiple-comparison test (95% confidence interval [CI], *P* < 0.0001). For the bactericidal assays, significance was evaluated using a one-way ANOVA with Tukey’s multiple-comparison test (95% CI, *P* < 0.0001). All calculations were performed on GraphPad Prism 9.2.0 (GraphPad).
