# Supplementary material for: FtlA and FtlB Are Candidates for Inclusion in a Next-Generation Multiantigen Subunit Vaccine for Lyme Disease
Source: Infect Immun. 2022 Sep 14;90(10):e00364-22. doi: 10.1128/iai.00364-22 (PMC9584329; doi:10.1128/iai.00364-22)
Supplement: Supplemental file 1 — Fig. S1 and S2 and Table S1. Download iai.00364-22-s0001.pdf, PDF file, 0.2 MB [file iai.00364-22-s0001.pdf]

Supplementary Figure 1. Ftl immunoblot analyses of Lyme disease isolates from Europe. Cell lysates of LD isolates from Europe (as labeled along the top) were fractionated by SDS-PAGE and the proteins stained with CBB (top panel) or transferred to PVDF membranes by immunoblotting. Identical immunoblots were screened with the anti-serum indicated to the left. All hyperimmune sera were used at a 1:1000 dilution. The migration positions of the MW standards are indicated in kDa. All immunoblots were imaged together for the same amount of time.

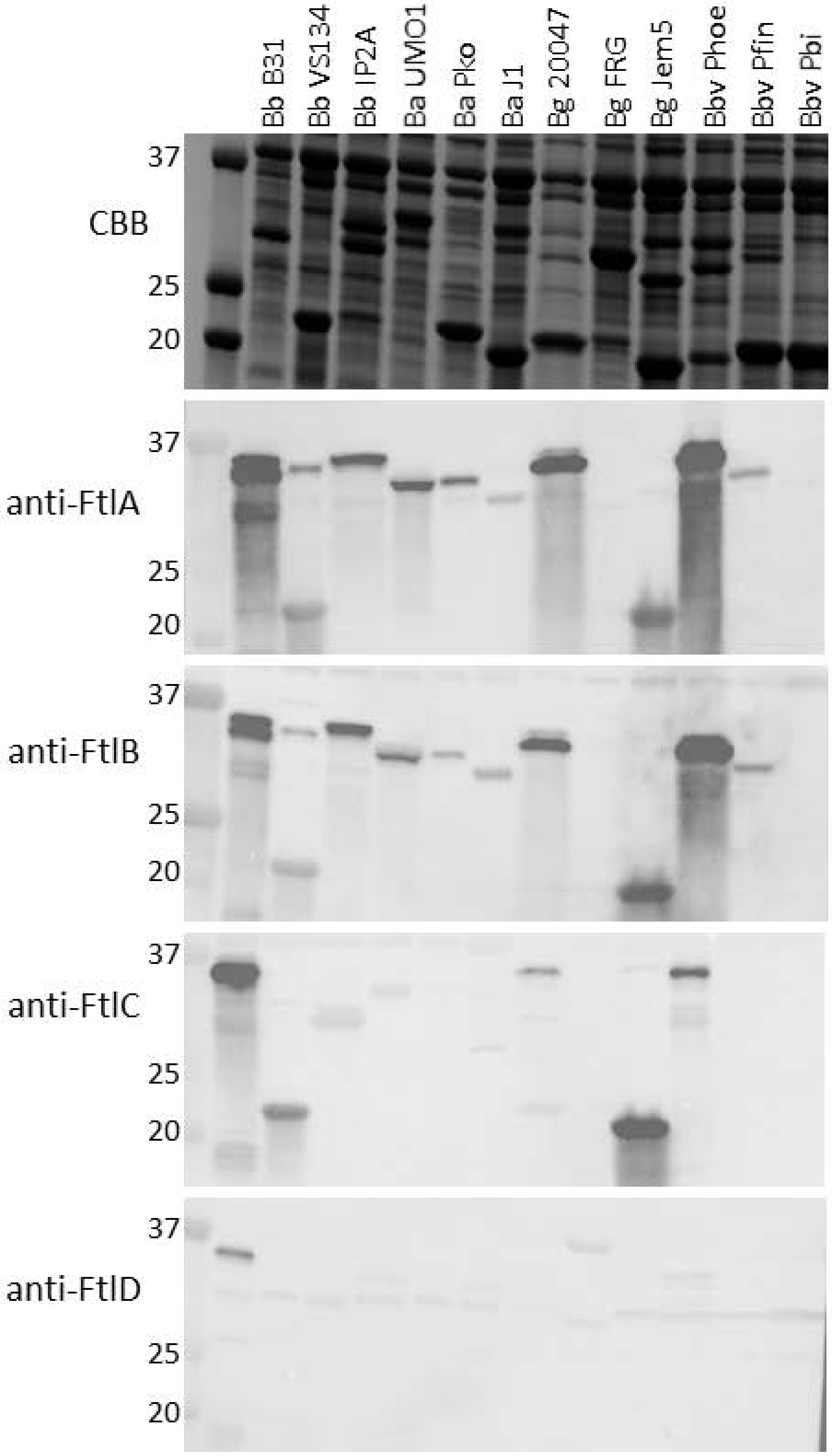

Supplementary Figure 1.

Supplementary Figure 2. Immunoblot screening for two different antigens (FtlA and BBK19) that are encoded by the same linear plasmid. *B. burgdorferi* cell lysates were fractionated by SDS-PAGE and the proteins stained with CBB (top panel) or transferred to PVDF membranes by immunoblotting. Identical immunoblots were screened with antiserum generated against two different proteins (FtlA and BBK19) that are encoded by genes carried by the same linear plasmid (*B. burgdorferi* B31; plasmid K; lp38) as indicated. The hyperimmune sera were used at a 1:1000 dilution. The migration positions of the MW standards are indicated in kDa. The immunoblots were imaged simultaneously.

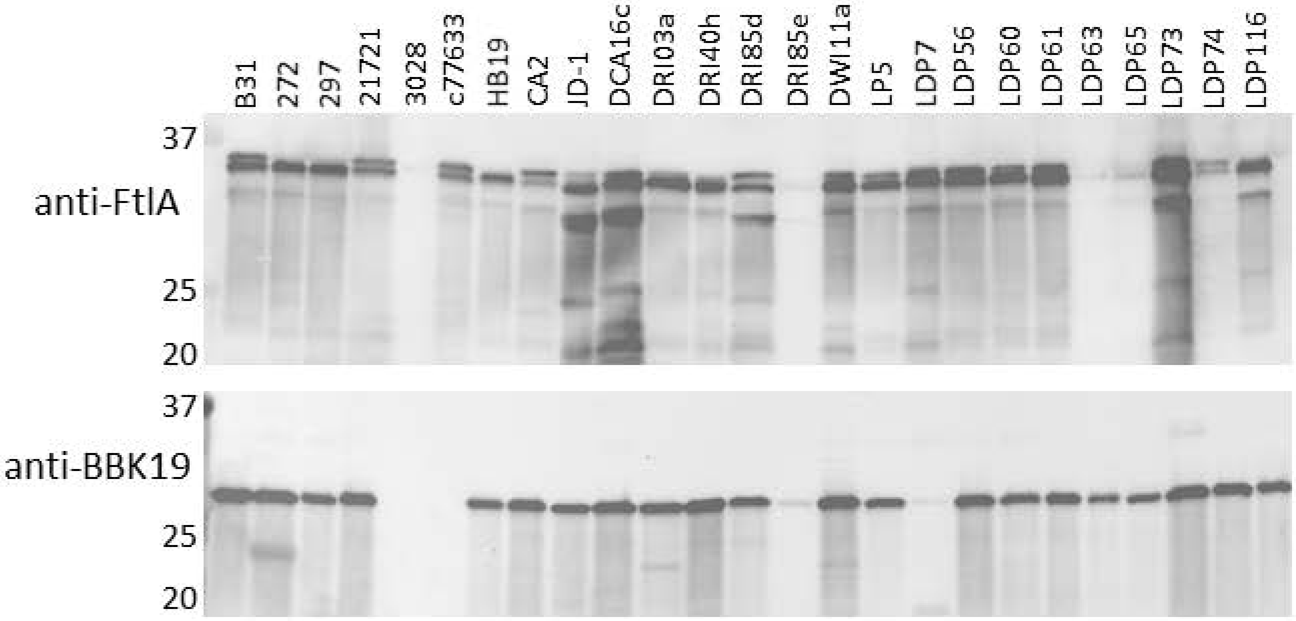

Supplementary Figure 2.

Supplementary Table 1.

Species/Isolate	Origin
B. burgdorferi B31	*I. scapularis;* NY, USA; clonal
B. burgdorferi 272	Human skin; NY, USA
B. burgdorferi 297	Human CSF; CT, USA; clonal
B. burgdorferi 21721	*I. scapularis*; WI, USA
B. burgdorferi 3028	Human skin; TX, USA
B. burgdorferi c77633	Mouse tissue; NY, USA; clonal
B. burgdorferi HB19	Human blood; CA, USA
B. burgdorferi CA2	*I. neotomae*; CA, USA
B. burgdorferi JD1	*I. scapularis*; NY, USA
B. burgdorferi LP5	Human skin; CT, USA
B. burgdorferi DCA16c	Skin biopsy isolate from a laboratory dog infected using field collected *I. pacificus* ticks from CA, USA; clonal
B. burgdorferi DRI103a, DRI140h, DRI185d, DRI185e	Skin biopsy isolates from laboratory dogs infected using field collected *I. scapularis* ticks from RI, USA; clonal
B. burgdorferi DWI11a	Skin biopsy isolate from a laboratory dog infected using field collected *I. scapularis* ticks from WI, USA; clonal
B. burgdorferi LDP7, LDP56, LDP60, LDP61, LDP63, LDP65, LDP73, LDP74, LDP116	Human blood isolates: MD, USA
B. burgdorferi VS134	*I. ricinus*; Switzerland
B. burgdorferi IP2a	Human CSF; France; clonal
B. afzelli UM01	Human skin; Sweden; clonal
B. afzelli Pko	Human skin; Germany; clonal
B. afzelli J1	*I. persulcatus*; Japan; clonal
B. garinii 20047	*I. ricinus*; France
B. garinii FRG	*I. ricinus*; Germany
B. garinii Jem5	Human skin; Japan
B. bavariensis PHoe, PFin, PBi	Human CSF; Germany

[a]Clonal populations were obtained by sub-surface plating.
